# HIV Infection and Testing among Latino Men Who Have Sex with Men in the United States: The Role of Location of Birth and Other Social Determinants

**DOI:** 10.1371/journal.pone.0073779

**Published:** 2013-09-03

**Authors:** Alexandra M. Oster, Kate Russell, Ryan E. Wiegand, Eduardo Valverde, David W. Forrest, Melissa Cribbin, Binh C. Le, Gabriela Paz-Bailey, NHBS Study Group

**Affiliations:** 1 Division of HIV/AIDS Prevention, Centers for Disease Control and Prevention, Atlanta, Georgia, United States of America; 2 University of Miami, Miami, Florida, United States of America; Johns Hopkins School of Public Health, United States of America

## Abstract

**Background:**

In the United States, Latino men who have sex with men (MSM) are disproportionately affected by HIV. Latino MSM are a diverse group who differ culturally based on their countries or regions of birth and their time in the United States. We assessed differences in HIV prevalence and testing among Latino MSM by location of birth, time since arrival, and other social determinants of health.

**Methods:**

For the 2008 National HIV Behavioral Surveillance System, a cross-sectional survey conducted in large US cities, MSM were interviewed and tested for HIV infection. We used generalized estimating equations to test associations between various factors and 1) prevalent HIV infection and 2) being tested for HIV infection in the past 12 months.

**Results:**

Among 1734 Latino MSM, HIV prevalence was 19%. In multivariable analysis, increasing age, low income, and gay identity were associated with HIV infection. Moreover, men who were U.S.-born or who arrived ≥5 years ago had significantly higher HIV prevalence than recent immigrants. Among men not reporting a previous positive HIV test, 63% had been tested for HIV infection in the past 12 months; recent testing was most strongly associated with having seen a health care provider and disclosing male-male attraction/sexual behavior to a health care provider.

**Conclusions:**

We identified several social determinants of health associated with HIV infection and testing among Latino MSM. Lower HIV prevalence among recent immigrants contrasts with higher prevalence among established immigrants and suggests a critical window of opportunity for HIV prevention, which should prioritize those with low income, who are at particular risk for HIV infection. Expanding health care utilization and encouraging communication with health care providers about sexual orientation may increase testing.

## Introduction

In the United States, Latino MSM are disproportionately affected by HIV infection.

The National HIV Behavioral Surveillance System (NHBS) reported that nearly one in five Latino MSM interviewed and tested in 2008 was infected with HIV [1]. Other studies have also found disproportionate rates of HIV infection among Latino MSM [2]–[4]. However, few studies have conducted within-group analyses to determine factors associated with HIV infection among Latino MSM.

In addition to understanding HIV prevalence, it is also important to assess HIV testing behavior. HIV testing and diagnosis allow persons to reduce transmission-related behavior and access HIV care and antiretroviral therapy that improve personal health and reduce transmission. Although NHBS data have shown that equal proportions of Latino, black, and white MSM have been tested for HIV in the past 12 months [1], barriers to HIV testing may be different for Latino MSM, and further analysis of NHBS data to explore factors associated with HIV testing among Latino MSM is warranted.

In recent years, increasing attention has been placed on monitoring the health of migrants [5], who face particular health-related challenges and barriers due to a variety of causes, including poverty and inadequate access to health services. In the United States, 37% of Latinos are foreign-born, and the population of foreign-born Latinos, which grew by 33% between 2000 and 2010 [6], is disproportionately affected by HIV infection [7]. Moreover, foreign-born Latino MSM are a diverse group who differ culturally based on their countries or regions of birth and their time in the United States; understanding these differences can help to target and tailor interventions appropriately.

Increasing evidence points to the fact that social determinants of health are important underlying causes of disease. Social determinants of health that affect population health include social environment (e.g., discrimination, ethnicity and cultural customs, income, and education), physical environment (e.g., place of residence, crowding), and health services (e.g., access to quality care) [8]. While several studies have explored the association of social determinants with HIV risk behavior among Latino MSM, few have examined associations with HIV infection or testing, and most studies of Latino MSM in the United States have been small or geographically limited.

NHBS is the largest and most geographically diverse surveillance system to monitor HIV risk among MSM in the United States. We used data from the second round of NHBS among MSM (NHBS-MSM2), conducted during 2008, to assess associations of location of birth and time since arrival as well as other social determinants of health with prevalent HIV infection and with recent HIV testing among Latino MSM in the United States.

## Methods

### National HIV Behavioral Surveillance System

NHBS-MSM2 was conducted in 21 metropolitan statistical areas (MSAs), selected based on a high number of people living with AIDS (Atlanta, Georgia; Baltimore, Maryland; Boston, Massachusetts; Chicago, Illinois; Dallas, Texas; Denver, Colorado; Detroit, Michigan; Houston, Texas; Los Angeles, California; Miami, Florida; Nassau, New York; Newark, New Jersey; New Orleans, Louisiana; New York City, New York; Philadelphia, Pennsylvania; San Diego, California; San Francisco, California; San Juan, Puerto Rico; Seattle, Washington; St. Louis, Missouri; and Washington, District of Columbia). MSM were recruited using venue-based, time-space sampling [9]. Activities included: 1) formative research to identify venues and times to recruit MSM [10]; 2) development of sampling frames of eligible venues and day-time periods; 3) random selection of venues and day-time periods; and 4) recruitment, interviewing, and testing during sampled events.

The eligibility criteria included being male, ≥18 years of age, a resident of the MSA, able to complete the survey in English or Spanish, and able to provide informed consent. Use of written vs. oral informed consent varied by site in accordance with local IRB requirements. Trained interviewers used handheld computers to administer a standardized questionnaire lasting approximately 30 minutes. Interviews consisted of questions about demographic characteristics, sexual and drug use behaviors, health insurance, use of health care and disclosure of male/male attraction or sexual behavior to a health care provider, HIV testing history, and use of HIV prevention services and programs. Participants were asked their country of birth and, if born outside the United States, the year of first arrival in the United States.

Anonymous HIV testing was offered to all participants regardless of self-reported HIV infection status. Local testing procedures varied by MSA, and consisted of collection of blood or oral specimens for either conventional laboratory testing or rapid testing in the field followed by laboratory confirmation. Participants received rapid test results and were given information on how to receive the results of laboratory-based testing.

### Ethics Statement

Activities for NHBS-MSM2 were approved by local IRBs for each of the 20 participating MSAs. All participants are explicitly assured during the recruitment process of the anonymous nature of the survey and the HIV testing. No personal identifiers are collected during enrollment, interview, or testing. All participants provide their informed consent to take part in the interview and to be tested for HIV. Because data collection is anonymous, participant names or other personal identifiers are not linked to any NHBS instruments.

### Inclusion Criteria

Participants were included in this analysis if they had a completed, valid survey; reported at least one male sex partner in the past 12 months; had a negative or confirmed positive HIV test result; and identified as Hispanic or Latino. Race/ethnicity was determined by two questions: first, a question about Hispanic or Latino ethnicity (“Do you consider yourself to be Hispanic or Latino?”), followed by a question about race, in which participants could select multiple races. Latinos could be of any race. Because we wanted to focus on Latino MSM living in the continental United States, we excluded data from San Juan, Puerto Rico, resulting in a sample from 20 MSAs.

### Statistical Analysis

We then described sociodemographic characteristics and risk/prevention behaviors of Latino MSM stratified by location of birth (United States [excluding Puerto Rico], Puerto Rico, Mexico, Caribbean [excluding Puerto Rico], Central America, or South America). For this analysis, we excluded foreign-born Latino MSM born in other locations (e.g., Europe, Asia). We also described characteristics of Latino MSM who were 1) U.S.-born, 2) foreign-born and arrived ≥5 years ago (established immigrants), and 3) foreign-born and arrrived <5 years ago (recent immigrants). For these analyses, men who resided in the continental United States but were born in Puerto Rico were considered foreign-born.

We calculated HIV prevalence among Latino MSM stratified by numerous characteristics. Then, after excluding the men who reported that they had previously tested positive for HIV infection, we calculated the proportion that had been tested for HIV infection in the past 12 months.

We also created models to determine associations between various factors and our two outcome variables (prevalent HIV infection and recent HIV testing). To control for clustering at the MSA level, we employed a generalized estimating equation-based Poisson model using a correction for a small number of clusters [11] in SAS PROC GLIMMIX [12]. We explored assuming a negative binomial distribution, but this did not improve the model fit. Hence, a Poisson distribution for the marginal model was used. Prevalence ratios are presented for unadjusted models and for multivariable models. Each multivariable model included age, education, income, sexual identity, and location of birth and time since arrival as predictor variables. We also tested models that included region of birth (rather than the location of birth and time since arrival variable). All tests and confidence intervals are two-sided and based on the 5% level of significance. SAS software [12] was used for all analyses. Data for the National HIV Behavioral Surveillance System are collected under a federal assurance of confidentiality and are not publicly available.

## Results

A total of 28,468 persons were approached for participation at 626 venues; 12,474 (44%) persons were screened for participation, 11,074 (89%) of whom were eligible for interview (81% of ineligible persons lived outside the MSA). Of these, 8153 (74%) consented to and completed both the interview and HIV test, had a valid test result, and reported male-male sex during the past 12 months. Among this group, 1734 (21%) were Latino MSM residing in the 20 metropolitan statistical areas in the continental United States. These 1734 men are included in this analysis.

Overall, median age was 31 years, and interquartile range was 24–40 years (Table 1). Over 60% had completed at least some college, and over one-third had a household income of <$20,000. Seventy-eight percent of men identified as gay or homosexual. Overall, 247 (14%) Latino MSM completed their surveys in Spanish. Of Latino MSM, 33% were recruited in bars, 28% in dance clubs, 10% in parks, beaches, and street locations, 9% in sex establishments, 6% in cafes, restaurants, and other retail establishments, 4% in social organizations, 3% at Gay Pride and other similar events, and 7% in other locations.

**Table 1:** Characteristics of Latino MSM by location of birth – 20 U.S. Cities, National HIV Behavioral Surveillance System, 2008.

	U.S.		Puerto Rico		Mexico		Caribbean		C. America		S. America			Total		
Characteristic	n	(%)	n	(%)	n	(%)	n	(%)	n	(%)	n	(%)		n	(%)	*P*
**Age (yrs)**																<.0001
Median (interquartile range)	29	(23–38)	34	(28–43)	31	(25–38)	41	(31–47)	30	(26–38)	35	(28–41)		31	(24–40)	
**Education**																<.0001
Less than high school graduate	83	(9)	16	(20)	39	(19)	19	(10)	29	(22)	4	(3)		190	(11)	
High school diploma or equivalent	261	(27)	21	(26)	66	(32)	57	(31)	41	(32)	36	(23)		485	(28)	
Some college or technical college	375	(39)	20	(24)	49	(24)	52	(29)	38	(29)	45	(29)		584	(34)	
College or higher education	243	(25)	25	(30)	53	(26)	53	(29)	21	(16)	69	(45)		475	(27)	
**Annual household income**																<.0001
0 to $19,999	283	(29)	39	(48)	93	(45)	83	(46)	60	(47)	39	(25)		599	(35)	
$20,000 to $39,999	258	(27)	15	(18)	70	(34)	66	(36)	40	(31)	59	(38)		517	(30)	
$40,000 to $74,999	256	(27)	17	(21)	36	(17)	24	(13)	19	(15)	39	(25)		395	(23)	
$75,000 or more	143	(15)	9	(11)	6	(3)	4	(2)	9	(7)	17	(11)		192	(11)	
**Employment status**																<.0001
Employed full-time	561	(58)	41	(50)	137	(66)	108	(60)	87	(67)	112	(73)		1061	(61)	
Employed part-time	155	(16)	12	(15)	43	(21)	22	(12)	17	(13)	20	(13)		271	(16)	
Unemployed	141	(15)	20	(24)	18	(9)	35	(19)	22	(17)	7	(5)		243	(14)	
Other	105	(11)	9	(11)	9	(4)	16	(9)	*	*	15	(10)		159	(9)	
**Number of years in United States**																<.0001
Mean	–	–	19		15		17.7		14		11.7			–	–	
Median	–	–	17		13.5		14		13		9			–	–	
Interquartile Range	–	–	10–26		8–19		6–28		7–20		6–17			–	–	
**Sexual identity**																<.0001
Gay or homosexual	766	(80)	53	(65)	174	(84)	141	(78)	79	(61)	127		(82)	1357	(78)	
Bisexual or heterosexual	195	(20)	29	(35)	33	(16)	39	(22)	50	(39)	27		(18)	375	(22)	
**Total**	962	(100)	82	(100)	207	(100)	181	(100)	129	(100)	154		(100)	1734	(100)	

Numbers may not add to total due to missing and 'unknown' responses. Persons born outside the listed regions (n = 19) are included in the total column only.

* Suppressed due to small cell size.

Almost half of Latino MSM had four or more partners in the past 12 months, and over half reported unprotected anal sex with a male partner in the past 12 months (Table 2). Sixteen percent had a female partner in the past 12 months and almost half reported non-injection drug use, but few (2%) reported injection drug use. Sixty percent had been tested for HIV in the past 12 months; of the 1368 men tested in the past 5 years, 795 (58%) had been tested in clinical settings, 463 (34%) in nonclinical settings, and 110 (8%) in settings that were not classifiable. Nearly three-quarters had visited a health care provider in the past 12 months, 66% reported telling a health care provider that they were attracted to or had sex with men, and 19% had received an individual or group-level behavioral HIV prevention intervention. Overall, 19% were HIV-positive; 11% tested positive and reported a previous positive test result and 8% tested positive but did not report a previous positive test result.

**Table 2:** Behaviors and HIV status of Latino MSM by location of birth – 20 U.S. Cities, National HIV Behavioral Surveillance System, 2008.

	U.S.		Puerto Rico		Mexico		Caribbean		C. America		S. America		Total		
Characteristic	n	(%)	n	(%)	n	(%)	n	(%)	n	(%)	n	(%)	n	(%)	*P*
**Number of male partners, past 12 mo**															0.2
1	233	(24)	22	(27)	47	(23)	32	(18)	35	(27)	23	(15)	396	(23)	
2	158	(16)	11	(13)	35	(17)	32	(18)	23	(18)	26	(17)	288	(17)	
3	137	(14)	15	(18)	26	(13)	24	(13)	20	(16)	16	(10)	242	(14)	
≥4	434	(45)	34	(41)	99	(48)	93	(51)	51	(40)	89	(58)	808	(47)	
**Unprotected anal sex with male, past 12 mo**															0.2
No	415	(43)	31	(38)	86	(42)	83	(46)	42	(33)	58	(38)	722	(42)	
Yes	547	(57)	51	(62)	121	(58)	98	(54)	87	(67)	96	(62)	1012	(58)	
**Female partner, past 12 mo**															<.0001
No	812	(84)	57	(70)	191	(92)	147	(81)	95	(74)	135	(88)	1454	(84)	
Yes	150	(16)	25	(30)	16	(8)	34	(19)	34	(26)	19	(12)	280	(16)	
**Non-injection drug use, past 12 mo**															<.0001
No	433	(45)	36	(44)	139	(67)	122	(67)	79	(61)	88	(57)	904	(52)	
Yes	528	(55)	46	(56)	68	(33)	59	(33)	50	(39)	66	(43)	829	(48)	
**Most recent HIV test**															0.002
Past 12 months	597	(62)	43	(52)	120	(58)	104	(57)	71	(55)	87	(56)	1036	(60)	
Greater than 1 year	276	(29)	31	(38)	54	(26)	65	(36)	38	(29)	59	(38)	528	(30)	
Never tested	86	(9)	8	(10)	32	(15)	12	(7)	20	(16)	8	(5)	166	(10)	
**Visited health care provider, past 12 mo**															<.0001
No	247	(26)	22	(27)	79	(38)	58	(32)	56	(43)	38	(25)	502	(29)	
Yes	715	(74)	59	(72)	128	(62)	123	(68)	73	(57)	116	(75)	1231	(71)	
**Ever told a health care provider they are attracted** **to or have sex with men**															<.0001
No	293	(30)	34	(41)	81	(39)	57	(31)	68	(53)	53	(34)	590	(34)	
Yes	666	(69)	48	(59)	126	(61)	124	(69)	61	(47)	99	(64)	1139	(66)	
**Received HIV behavioral intervention, past 12 mo**															0.004
No	777	(81)	65	(79)	150	(72)	155	(86)	114	(88)	121	(79)	1398	(81)	
Yes	185	(19)	17	(21)	57	(28)	26	(14)	15	(12)	33	(21)	336	(19)	
**NHBS HIV test results**															0.009
HIV-positive total	158	(16)	23	(28)	35	(17)	41	(23)	22	(17)	41	(27)	322	(19)	
HIV-positive and self-reported positive	90	(9)	15	(18)	20	(10)	24	(13)	8	(6)	28	(18)	185	(11)	
HIV-positive and not self-reported positive	68	(7)	8	(10)	15	(7)	17	(9)	14	(11)	13	(8)	137	(8)	
HIV-negative	804	(84)	59	(72)	172	(83)	140	(77)	107	(83)	113	(73)	1412	(81)	
**Total**	962	(100)	82	(100)	207	(100)	181	(100)	129	(100)	154	(100)	1734	(100)	

Numbers may not add to total due to missing and 'unknown' responses. Persons born outside the listed regions (n = 19) are included in the total column only.

Of the 1734 Latino MSM included in this analysis, 962 (55%) were born in the United States (excluding Puerto Rico), 82 (5%) in Puerto Rico, 207 (12%) in Mexico, 181 (10%) in the Caribbean (excluding Puerto Rico), 129 (7%) in Central America, 154 (9%) in South America, and 19 (1%) in other regions of the world. Median time since arrival for foreign-born men was 13 years. Tables 1 and 2 present the characteristics of men by location of birth. In general, men born in Puerto Rico and Central America more commonly identified as bisexual or heterosexual and had female partners than men born elsewhere. Men born in Central America also more commonly reported unprotected anal sex. Men born in Central America less commonly reported visiting a health care provider in the past 12 months or ever disclosing male-male sexual attraction or behavior to a health care provider. Men born in Puerto Rico more commonly reported injection drug use (11% vs. 2% overall).

**Figure 1:**
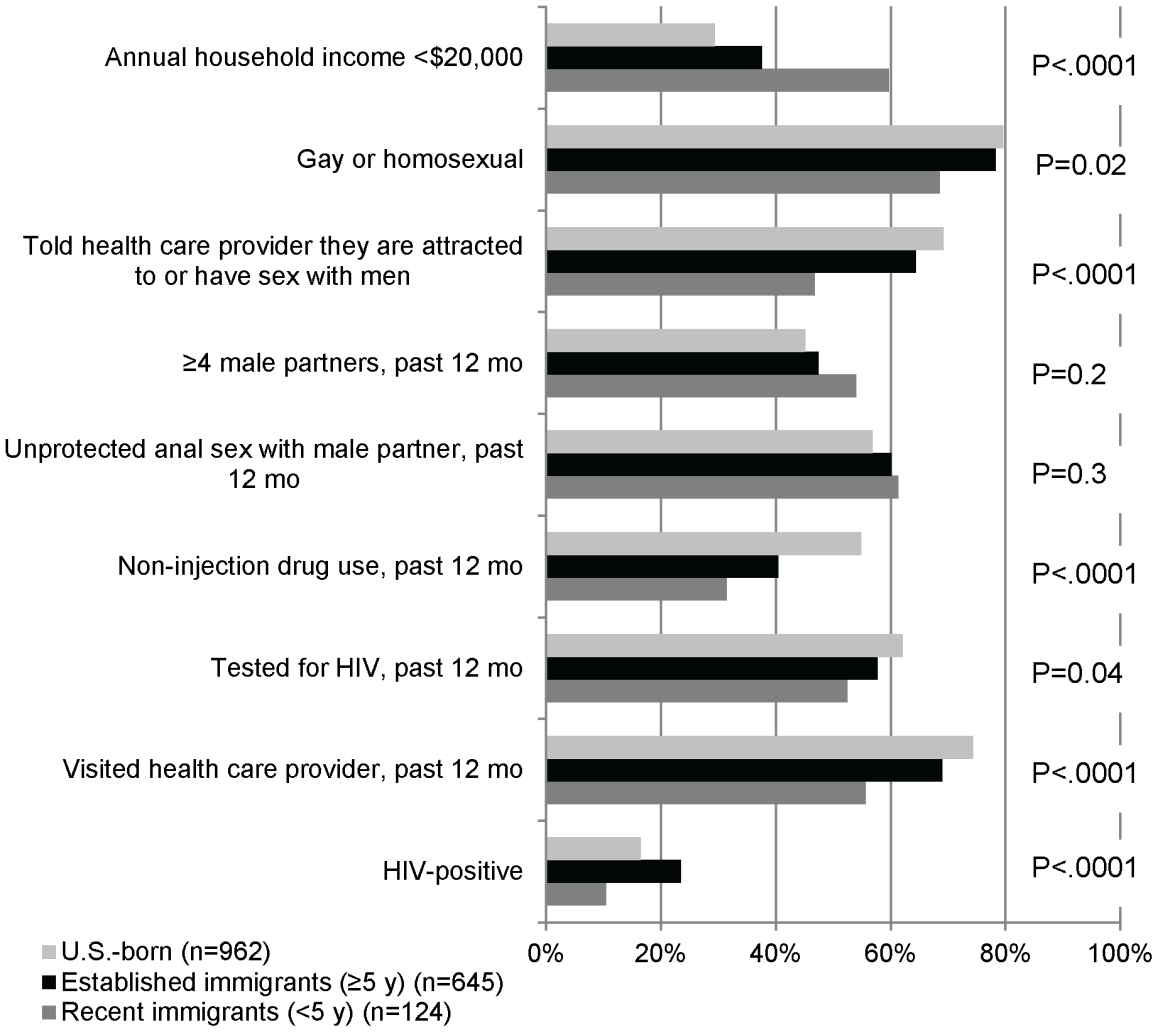
Characteristics of Latino MSM by location of birth and time since arrival, 20 U.S. cities.

Figure 1 presents characteristics of Latino MSM stratified by whether they were U.S.-born, established immigrants, or recent immigrants. Recent immigrants had lower income, less commonly identified as gay, and were less likely to tell a health care provider they were attracted to or had sex with men. They more commonly reported 4 or more male partners, but less commonly reported non-injection drug use. Fewer recent immigrants had been tested for HIV in the past 12 months or had visited a health care provider. Recent immigrants also had lower HIV prevalence than established immigrants or U.S.-born Latino MSM.

Overall, HIV prevalence among Latino MSM was 19% (Table 3). In multivariable analysis adjusting for education, prevalent HIV infection was associated with older age, lower household income, gay identity, and being U.S.-born or an established immigrant, both compared with being a recent immigrant. In a multivariable model that included region of birth (United States, Puerto Rico, Mexico, Caribbean, Central America, South America, or other) instead of the combined variable on location of birth and time since arrival, region of birth was not significantly associated with HIV infection (data not presented).

**Table 3:** HIV prevalence among Latino MSM by selected characteristics and adjusted risk of prevalent infection – 20 U.S. Cities, National HIV Behavioral Surveillance System, 2008*.

		HIV-positive		Adjusted Prevalence Ratio*	95% CI
Characteristic	Total	n	(%)		
**Age (yrs)**				**1.5 per 10 y increase**	**1.3–1.7**
18–19	100	4	(4)		
20–24	345	30	(9)		
25–29	336	43	(13)		
30–39	513	113	(22)		
40–49	342	98	(29)		
≥50	98	34	(35)		
**Education**					
Less than high school graduate	190	43	(23)	1.4	0.7–2.7
High school diploma or equivalent	485	84	(17)	1.1	0.8–1.6
Some college or technical college	584	115	(20)	1.2	0.8–1.9
College or higher education	475	80	(17)	Referent	
**Annual household income**					
0 to $19,999	599	140	(23)	**1.8**	**1.1–2.9**
$20,000 to $39,999	517	96	(19)	1.4	0.9–2.1
$40,000 to $74,999	395	53	(13)	1.0	0.7–1.4
$75,000 or more	192	29	(15)	Referent	
**Sexual identity**					
Gay or homosexual	1357	274	(20)	**2.4**	**1.2–4.7**
Bisexual	353	44	(12)	1.4	0.6–3.2
Straight or heterosexual	22	3	(14)	Referent	
**Location of birth and time since arrival**					
U.S.-born	962	158	(16)	**1.6**	**1.1–2.4**
Foreign-born, arrived ≥5 y ago	645	151	(23)	**1.7**	**1.2–2.6**
Foreign-born, arrived <5 y ago	124	13	(10)	Referent	
**Total**	1734	322	(19)		

Numbers may not add to total due to missing and 'unknown' responses.

* Model includes all of the variables listed and also accounts for clustering at the level of the MSA. Statistically significant findings are shown in bold font.

After excluding 185 participants who reported a previous positive HIV test, we conducted an analysis of recent HIV testing among the remaining 1549 MSM. Of these MSM, 63% had been tested for HIV infection in the past 12 months (Table 4). In multivariable analysis adjusting for household income, sexual identity, and health insurance, recent HIV testing was associated with younger age, visiting a health care provider in the past 12 months, and telling a health care provider that they are attracted to or have sex with men. Recent HIV testing was less common among men with a high school diploma or equivalent, compared with those with college or higher education. Recent HIV testing was not associated with location of birth and time since arrival. As was the case with the model for prevalent HIV infection, in a model that substituted region of birth for the combined variable on location of birth and time since arrival, region of birth was not significantly associated with recent HIV testing (data not presented).

**Table 4:** Recent HIV testing among Latino MSM and adjusted prevalence of testing – 20 U.S. Cities, National HIV Behavioral Surveillance System, 2008*.

	Total	HIV test in past 12 months		AdjustedPrevalence Ratio**	95% CI
Characteristic		n	(%)		
**Age (yrs)**				**0.9 per 10y increase**	**0.8–0.9**
18–19	99	70	(71)		
20–24	336	220	(65)		
25–29	315	207	(66)		
30–39	446	295	(66)		
40–49	283	146	(52)		
>50	70	33	(47)		
**Education**					
Less than high school graduate	169	87	(51)	0.8	0.7–1.01
High school diploma or equivalent	441	255	(58)	**0.9**	**0.8–0.9**
Some college or technical college	512	331	(65)	0.9	0.8–1.01
College or higher education	427	298	(70)	Referent	
**Annual household income**					
0 to $19,999	512	281	(55)	1.0	0.8–1.1
$20,000 to $39,999	467	304	(65)	1.0	0.9–1.2
$40,000 to $74,999	368	242	(66)	1.0	0.9–1.1
$75,000 or more	171	119	(70)	Referent	
**Sexual identity**					
Gay or homosexual	1188	772	(65)	1.1	0.7–1.8
Bisexual	340	190	(56)	1.0	0.6–1.7
Straight or heterosexual	20	8	(40)	Referent	
**Location of birth and time since arrival**					
U.S.-born	872	565	(65)	1.1	0.9–1.3
Foreign-born, arrived ≥5 y ago	555	343	(62)	1.2	0.97–1.4
Foreign-born, arrived <5 y ago	119	61	(51)	Referent	
**Health insurance**					
Public	692	474	(68)	1.0	0.9–1.2
Private	114	74	(65)	1	0.96–1.1
Other/multiple	15	12	(80)	1.1	0.9–1.5
None	715	403	(56)	Referent	
**Visited health care provider, past 12 mo**					
Yes	1056	759	(72)	**1.6**	**1.3–1.8**
No	492	211	(43)	Referent	
**Told health care provider they are attracted to or have sex with men**					
Yes	970	681	(70)	**1.3**	**1.2–1.3**
No	574	285	(50)	Referent	
**Total**	**1549**	**971**	(**63**)		

Numbers may not add to total due to missing and 'unknown' responses.

* Participants who reported previously testing positive for HIV infection were excluded.

** Model includes all of the variables listed and also accounts for clustering at the level of the metropolitan statistical area. Statistically significant findings are shown in bold font.

Of those who had not been tested in the past 12 months, the two most frequently cited main reasons for not being tested were believing oneself to be at low risk (32%) and being afraid of finding out that one had HIV (31%). However, when limited to those who tested positive, 40% cited being afraid of finding out that one had HIV as their main reason, and an additional 13% cited other reasons that were potentially related to HIV stigma, including that they were worried someone would find out the results, they were worried their name would be reported to the government, or that they were worried they would lost their job, insurance, or their house.

## Discussion

We found that, among Latino MSM, HIV infection was associated with location of birth and time since arrival as well as income. We also found that men with limited education and men who had not visited a health care provider in the past 12 months were less likely to be recently tested for HIV. Moreover, men who had not told a health care provider they were attracted to or had sex with men were less likely to report recent testing, and many of those who were not tested reported reasons that were likely related to fear of HIV or HIV stigma. Each of these findings highlights the important role of social determinants of health and has potential implications for targeting and delivery of HIV prevention interventions.

Compared with recent immigrants, established immigrants and U.S.-born MSM had higher HIV prevalence even after adjusting for age, income, education, and sexual identity. This finding suggests that many foreign-born MSM acquire HIV after arrival and indicates the presence of a critical window of opportunity for HIV prevention for recent immigrants. Qualitative interviews with migrant Latino MSM in New York City have indicated that many Latino MSM immigrated to the United States to escape social environments in their countries of origin that were hostile toward MSM and that arrival in the United States often leads to increased sexual freedom and disinhibition [13]. These findings may explain why duration of residency has been found to be associated with HIV risk behavior and HIV infection among Latino MSM in South Florida, with those present for less than one year having the lowest levels of both [14]. Combining findings from our analysis with the fact that foreign-born Latinos with shorter durations in the United States have been found to have significantly lower levels of HIV/AIDS knowledge suggests that efforts to reach this group may be particularly important [15].

HIV infection among Latino MSM was also associated with low income, and those with lower education were less likely to report a recent HIV test. Others have found that financial hardship is associated with risky sexual behavior among Latino MSM [16]. Although our analysis does not explain the exact link between income and HIV risk or between education and HIV testing, it suggests that public health officials should make special efforts to reach Latino MSM of low socioeconomic status with HIV prevention efforts and that we should work to improve access of this population to care and testing.

Recent HIV testing was most highly associated with visiting a health care provider in the past 12 months and disclosing male-male attraction or sexual behavior to a health care provider. This, in combination with the high proportion who had been tested in a clinical setting, which has also been found previously [17], suggests an important role for testing in clinical settings. In the NHBS sample, Latino MSM were less likely than white MSM to disclose male-male attraction or sexual behavior to a health care provider (data not presented); this may be related to strong cultural beliefs, such as machismo, that stigmatize sexual attraction to other men, although NHBS does not directly measure these beliefs [18], [19]. Additionally, Latino MSM may face the possibility of discrimination due to not only homophobia, but also racism and classism [16]; the combination of these factors may further complicate men’s decisions to disclose this important information to health care providers. In communities where these factors may encourage secrecy with respect to male-male sexual behavior, sensitivity training for health care providers and staff at HIV testing sites should be strongly encouraged and promoted.

We also found that many of those who had not recently been tested but tested positive during NHBS reported that they had not been tested for reasons that may be related to HIV stigma. HIV stigma is prevalent in Latino culture [18], and HIV stigma and homophobia are often closely linked, with the public often equating HIV and same-sex behavior [20]. HIV stigma has also been linked to reluctance to access health care, be tested for HIV, and to disclose positive HIV status [18], [21], [22].

The findings from this analysis have helped shape CDC’s REASONS/RAZONES mass media campaign to encourage HIV testing among Latino MSM (http://hivtest.cdc.gov/reasons/). This campaign, which launched in June 2013, features positive portrayals of Latino MSM, normalizes HIV testing through positive messages, and includes messages to reach those with lower education, lower literacy, and varying levels of acculturation.

In descriptive analysis, HIV prevalence and HIV testing varied substantially by region of birth, with prevalence highest among those born in Puerto Rico, South America, and the Caribbean, and testing lowest among those born in Puerto Rico and Central America. These differences, which did not persist on multivariable analysis, may be due to confounding by other characteristics of the interviewed populations, such as age (for prevalence) or disclosure of attraction to/sex with men to a health care provider (for testing). The differences in risk behaviors, HIV testing and coverage of prevention interventions by region of birth may be useful information when designing prevention strategies in cities with large Latino populations.

### Limitations

This analysis is subject to several limitations. First, these data are not weighted and are not necessarily representative of all Latino MSM in the participating MSAs. For example, non-gay-identified men may have been less likely to attend recruitment venues. However, this is a large sample of MSM and we present data from substantial numbers of MSM from many regions of the world. Social desirability bias may have resulted in overestimates of the proportion tested and underestimates of the number who previously tested positive for HIV infection. We do not have data on preferred language or level of acculturation and therefore cannot address how acculturation may play into HIV risk and prevention. Moreover, we did not have information about desire to remain in the United States vs. returning to one’s home country; therefore, we do not know which recent immigrants will remain and become established immigrants. Finally, the immigration policy that banned entry to the United States for HIV-positive persons was lifted in 2009, after these data were collected; we are not able to address the effect of this ban.

## Conclusions

Efforts to understand the epidemiology of HIV infection, and particularly disparities in HIV infection, are contingent on understanding the underlying determinants of risk [23]. The World Health Organization Commission on Social Determinants of Health concluded that the most important determinants of one’s health status are the social conditions in which people are born, live, and work [24]. This analysis contributes to the growing body of literature on the importance of social determinants of health for HIV risk. To be successful, efforts to achieve health equity and reduce HIV incidence must address these proximal determinants of risk. Given the findings of our analysis, efforts to reduce HIV incidence among Latino MSM must address access to and utilization of health care, homophobia and HIV-related stigma, and poverty. Doing so will require taking an interdisciplinary approach and collaborating across a wide range of disciplines [25]. While these interventions are often the most difficult to implement, they also have the potential for the greatest impact on health [26].
